# Analysis of Pleasure and Displeasure in Harmony Between Colored Light and Fragrance by the Left and Right OFC Response Differences

**DOI:** 10.3390/s25072230

**Published:** 2025-04-02

**Authors:** Toshinori Oba, Midori Tanaka, Takahiko Horiuchi

**Affiliations:** 1 Graduate School of Science and Engineering, Chiba University, Chiba 263-8522, Japan; 2 Graduate School of Informatics, Chiba University, Chiba 263-8522, Japan

**Keywords:** crossmodal perception, olfactory, color, harmony, emotion, orbitofrontal cortex, near-infrared spectroscopy

## Abstract

**Highlights:**

**What are the main findings?**

- By sensing biological signals, we have experimentally revealed that the left OFC is activated when perceiving a harmonious combination of colored light and fragrance, whereas the right OFC is activated when the combination is disharmonious.
- By sensing biological signals, we have experimentally revealed that functional differences between the left and right OFC are influenced not only by the pleasure–displeasure axis but also by the crossmodal–multimodal perception axis.

**What is the implication of the main finding?**

- We can develop a scientific understanding of the emotional impact of visual and olfactory interactions based on biological information sensing.
- Our study lays the groundwork for further developments in sensor-based neuroscience and biomedical engineering.

**Abstract:**

Daily actions are influenced by sensory information. Several studies have investigated the multisensory integration of multiple sensory modalities, known as crossmodal perception. Recently, visual–olfactory crossmodal perception has been studied using objective physiological measures rather than subjective evaluations. This study focused on sensing in the orbitofrontal cortex (OFC), which responds to visual and olfactory stimuli, and may serve as a physiological indicator of perception. Using near-infrared spectroscopy (NIRS), we analyzed the emotions evoked by combinations of colored light and fragrance with a particular focus on the lateralization of brain function. We selected pleasant and unpleasant fragrances from some essential oils, paired with colored lights that were perceived as either harmonious or disharmonious with the fragrances. NIRS measurements were conducted under the four following conditions: fragrance-only, colored light-only, harmonious crossmodal, and disharmonious crossmodal presentations. The results showed that the left OFC was activated during the crossmodal presentation of a harmonious color with a pleasant fragrance, thereby evoking pleasant emotions. In contrast, during the crossmodal presentation of a disharmonious color with an unpleasant fragrance, the right OFC was activated, suggesting increased displeasure. Additionally, the lateralization of brain function between the left and right OFC may be influenced by ‘pleasure–displeasure ’ and ‘crossmodal perception–multimodal perception’.

## 1. Introduction

We obtain various types of information every day using the five senses and select appropriate actions in response. For example, we can simultaneously perceive multiple sensory inputs such as ‘video and sound’ or ‘color and scent’. We then integrate this information to select appropriate actions. While the five senses have been investigated in studies from various domains, the number of studies on multisensory integration, also referred to as crossmodal or multimodal integration, has increased in recent years [1]. Both crossmodal and multimodal are terms related to multiple modalities, but they differ in meaning and usage. Crossmodal generally refers to situations where multisensory integration occurs through interactions between different modalities. Here, “interaction” means that two or more perceptual stimuli influence each other. Specifically, this includes cases where a stimulus in one modality affects the perception or cognition of another modality, as well as instances where such influences occur bidirectionally. On the other hand, multimodal refers to situations where multiple modalities coexist and are integrated without necessarily interacting with each other. In other words, the key difference between crossmodal and multimodal integration lies in whether the modalities influence one another during integration or remain independent. This study analyzes crossmodal perception, where interactions between different modalities influence perception.

Traditional crossmodal study has primarily focused on vision [2]. However, in recent decades, crossmodal interactions involving other senses, such as olfaction, have also been discussed. Even within the scope of visual–olfactory crossmodal perception, numerous studies have been reported [3,4,5,6,7,8,9,10,11,12,13,14,15,16,17]. Zellner et al. [3] focused on the visual–olfactory crossmodal perception and investigated the effect of color on the perceived intensities of specific food scents. Their experiment involved participants evaluating the intensity of scents in a red liquid and a colorless liquid, each containing a strawberry scent. The results indicated a tendency for the colored liquid to be perceived as having a stronger scent than the colorless liquid. Gilbert et al. [4] confirmed significant color associations for 20 different fragrances that remained stable, even when the experiment was repeated years later, suggesting a strong correlation between vision and olfaction. Miura et al. [5] used color patches to investigate the effects of color–fragrance harmony on stress reduction. They found that harmonious color–fragrance combinations reduce stress more than disharmonious combinations. In addition, some studies have focused on the speeds of processing and discriminating scent. Demattè et al. [6] presented participants with scents and color patches and instructed them to discriminate each stimulus as quickly and accurately as possible. The stimuli were presented under either a compatible condition (e.g., a strawberry scent with pink or a spearmint scent with turquoise) or an incompatible condition (e.g., a strawberry scent with turquoise or a spearmint scent with pink). Reaction times and identification accuracy were measured. The results showed that the more the color and scent were matched, the faster and more accurate the responses became. In addition, Demattè et al. [7] showed that when the color and shape are congruent with a scent, the accuracy of scent discrimination increases, whereas incongruence leads to a decrease in accuracy. Various studies have demonstrated the existence of crossmodal associations between vision and olfaction.

While previous studies have relied primarily on subjective evaluations, recent studies have increasingly focused on objective analyses using physiological responses [8,9,10,11,12,13,14,15,16,17]. Österbauer et al. [8] investigated the neurophysiological correlates of visual–olfactory crossmodal perception using functional magnetic resonance imaging (fMRI). Their experiment showed that the orbitofrontal cortex (OFC) and the caudal region of the insular cortex exhibited greater activation as the congruence between scent and color pairs increased. Furthermore, numerous studies have measured physiological responses using near-infrared spectroscopy (NIRS), which has a higher temporal resolution than fMRI [9,10,11,12,13,14,15,16,17]. Compared to existing neuroimaging techniques, NIRS is relatively inexpensive, easy to set up, and does not require a specialized environment. Its high motion tolerance allows studies to be conducted in natural settings. Pinti et al. [9] stated that NIRS allows the study of visual–olfactory crossmodal perception in natural environments, generating more reliable and ecologically valid data. NIRS measures brain activity at a maximum depth approximately halfway between the light source and detector [10]. The maximum distance between the light source and detector to maintain detectable signals was 3 cm, resulting in a measurement depth of approximately 1.5 cm from the scalp surface. Therefore, it is challenging to use NIRS to measure the deep brain regions associated with olfactory perception, such as the piriform cortex, insular cortex, and hippocampus [11]. However, among the regions associated with olfactory and visual perception, there are relatively shallow areas, such as the OFC, which have been investigated by recent studies using NIRS [12,13].

The connection between colored light and olfactory sensing is an emerging topic with important implications for sensor-based research and applications. Our study contributes to this area by demonstrating how NIRS can be utilized to detect the neural responses associated with multisensory integration. Understanding these interactions is crucial for the following reasons:

1. Advancements in Sensory Monitoring Technologies

Investigating how different sensory modalities interact provides essential insights for developing advanced sensor systems. For example, our findings could inform the design of multimodal sensing technologies used in healthcare, smart environments, and human-computer interaction.

2. Enhancing Sensor-Based Brain Activity Monitoring

By applying NIRS to study multisensory interactions, our work contributes to refining non-invasive brain sensing methodologies. These insights could support further developments in real-time brain monitoring, particularly in areas like neurofeedback, assistive technologies, and affective computing.

In previous studies on visual–olfactory crossmodal perception, various types of colors have been used as visual stimuli, such as colored liquids [3,14], color patches [4,5,6,7], and monitor displays [16], all of which are small-scale visual stimuli of a few centimeters by a few centimeters, focusing on flat visual stimuli. However, since we live in environments with ambient lighting daily, spatial testing is important. Yamashita et al. [16] and Oba et al. [17] used physiological indicators to analyze the color–fragrance harmony in lighting environments. Colored light was used as the color stimulus for spatial examination, and an objective analysis was performed by measuring the OFC response to the stimuli. Yamashita et al. [16] initially conducted a preliminary survey using five fragrances and 36 colored lights to select a ‘pleasant fragrance’ and ‘unpleasant fragrance’, as well as ‘harmonious colors’ and ‘disharmonious colors’ for each participant. The OFC response was measured under the four following conditions: (1) fragrance-only, (2) colored light-only, (3) harmonious crossmodal, and (4) disharmonious crossmodal presentations. The results showed that the highest OFC activation occurred during the fragrance-only presentation, with the left OFC showing greater activation than the right OFC. This suggested a relationship between pleasant emotions and the left OFC. Furthermore, Oba et al. [17] analyzed whether differences in the lighting environment affected OFC activation and whether the left and right OFC activated differently based on pleasure or displeasure. They found that the OFC was more activated in a relaxing lighting environment closer to real-life conditions. Moreover, pleasant emotions were associated with greater activation of the left OFC, which supported the results of Yamashita et al. [16]. However, these studies used a limited number of fragrances as stimuli, and these were generally perceived as pleasant by the participants. Therefore, the analysis focused on pleasant emotions related to the left OFC, making it challenging to analyze unpleasant emotions. This study aimed to analyze the pleasure–displeasure emotions evoked by color–fragrance harmony while also considering unpleasant emotions based on physiological indicators and focusing on the lateralization of brain function.

Our work contributes to the sensing field in two key ways:

1. Application of NIRS in Multisensory Research

We demonstrated the effectiveness of NIRS as a non-invasive tool to measure cortical activity related to the interaction between light and olfactory stimuli. This application showcases the potential of NIRS in advancing real-time sensory monitoring, expanding its use beyond traditional domains.

2. Novelty in Sensing Approach

Although we did not develop a new sensor, our methodological approach integrates NIRS into a novel approach to explore sensory processing. Specifically, we applied NIRS to capture the subtle hemodynamic responses associated with crossmodal interactions, which could inspire further advancements in sensor-based neuroscience research.

## 2. Experiment I: Analysis of Pleasure and Displeasure in Harmony of Colored Light and Fragrance

### 2.1. Measurement of Orbitofrontal Cortex Responses

Recent psychophysical experiments dealing with emotions have increased the use of physiological indicators such as brain waves for objective analysis. In previous studies [9,10,11,12,13,14,15,16,17], the OFC was used as a physiological indicator. The OFC is located on the ventral surface of the prefrontal cortex and is associated with the integration of sensory information, memory, and decision-making [18]. More specifically, it plays an important role in regulating the cognitive and social behaviors related to rewards and punishments [19]. Additionally, the activity of the OFC has been shown to correlate with the pleasure or displeasure evoked by visual and olfactory stimuli, with activation of regions near the OFC when these emotions are evoked [20,21]. Furthermore, the left OFC is correlated with positive emotions, such as pleasure, while the right OFC is associated with negative emotions, such as displeasure, and a hemispheric difference in OFC responses has been reported [22]. Based on these previous studies, OFC responses could be considered physiological indicators for analyzing psychophysical experiments focusing on crossmodal perception between colors and fragrances.

NIRS is a non-invasive method that measures changes in the oxygenated hemoglobin (oxyHb) concentration using near-infrared light on biological tissues. This technique is particularly useful for measuring brain activity and is widely used in cognitive neuroscience and medical fields [23]. Near-infrared light is able to penetrate biological tissues such as the scalp, skull, and brain tissue. Additionally, hemoglobin in the blood has different absorption characteristics depending on its oxidation state. Based on these properties, light emitted from a source passes through the tissue, undergoes scattering and absorption within the body, and the reflected light is detected by a detector. The change in the intensity of the light is measured. This intensity change is based on the Beer–Lambert law. Another method for measuring brain activity is fMRI, which provides high spatial resolution by imaging not only structural information but also functional activity in the brain. However, fMRI requires large equipment and it is restrictive, as subject movement is not allowed. In contrast, NIRS, although having a lower spatial resolution than fMRI, has a low constraint, and brain function can be measured in more natural states or under light physical activities without restrictions on the participant’s posture. This makes NIRS a suitable method for the psychophysical experiments in this study. Therefore, in this study, we attempted to measure OFC responses using NIRS. During the experiment, NIRS (Hb133, ASTEM Co., Ltd., Kawasaki, Japan) was used to measure the oxyHb concentration changes at two locations above the eyebrows on the left and right sides (channel 1, near the left OFC, and channel 2, near the right OFC). The NIRS plate was positioned immediately above the eyebrows and symmetrically aligned. The total length of the plate was 14 cm, with the light source on the outside and the receiver placed on the inside. The measurement point corresponds to a 4 cm offset to the left or right from the center of the forehead. There are two channels, and they are not positioned in 3D. The sampling frequency of the measurement was 10 Hz, and during data analysis, a moving average was applied to the raw data. Changes in oxyHb concentrations were measured using the software supplied with Hb133. From this data, the difference in the peak values of the oxyHb concentrations before and during stimulus presentation (ΔoxyHb) was calculated, and this value was used as the response to the stimulus for analysis. The ΔoxyHb was calculated by the authors’ own program based on those measurements.

### 2.2. Generation of Experimental Stimuli

**Figure 1:**
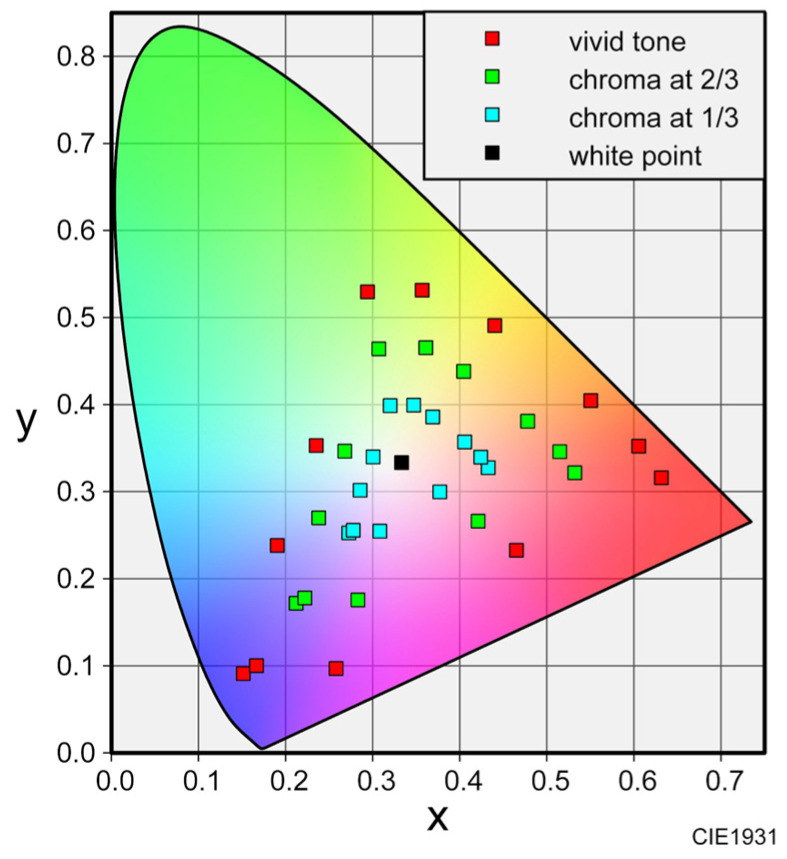
Chromaticity coordinates of the 36 generated colored lights.

First, we generated colored lights that served as visual stimuli for the participants. The colors used in the experiment were determined using a practical color coordinate system (PCCS), a two-dimensional system based on hue and tone that is suitable for considering color harmony. We referred to the vivid tone, which is the highest chroma tone in the PCCS, and selected 12 hues at equal intervals from the 24 vivid tones. Additionally, to ensure a sufficient examination of the colors that harmonize with fragrances, we prepared more colors by setting the chroma of the pure colors to two-thirds and one-third, respectively, based on white light (x = 0.33, y = 0.33). Consequently, we generated 36 colored lights by considering three chroma levels of the 12 hues. The chromaticity coordinates of the colored light are shown in Figure 1.

Next, we generated fragrances that served as olfactory stimuli. We used essential oils that are considered suitable for eliciting physiological responses, owing to their ability to emit strong fragrances in small amounts. The five fragrances used by Yamashita et al. [16] and Oba et al. [17]—vanilla, hinoki, mint, lime, and grapefruit—were relatively pleasant to the participants. In this study, we increased the variety of fragrances and analyzed the emotions evoked by unpleasant fragrances as well. We used 12 types of fragrances, including those used by Yamashita et al. [16] and Oba et al. [17], as well as unpleasant fragrances. The 12 fragrances used in the preliminary experiment were ylang-ylang, orange, ginger, patchouli, vanilla, palmarosa, hinoki, mint, lime, ravensara, lavender, and lemon. To minimize individual differences in the perception of pleasure or displeasure, we considered various categories such as citrus, floral, and resinous fragrances. Essential oils were absorbed on 1 cm^2^ cotton pads and placed in 20 mL amber glass bottles. It was difficult to determine the color of the essential oils absorbed in these bottles. Olfactory stimuli were presented using the ‘odor bottle method’, in which participants brought the bottle close to their nose to smell the fragrance. The advantage of this method is that the bottles are lightweight and easy to hold, making it easy to present stimuli to participants and minimize movement, thereby reducing the noise in the NIRS measurements. Furthermore, we ensured that all fragrances had the same intensity by using a fragrance meter (OMX-SRM, Shinyei Technology Co., Ltd., Kobe, Japan) to adjust the fragrance intensity near the opening of the bottle to 100.

### 2.3. Selection Survey for Experimental Stimuli

**Figure 2:**
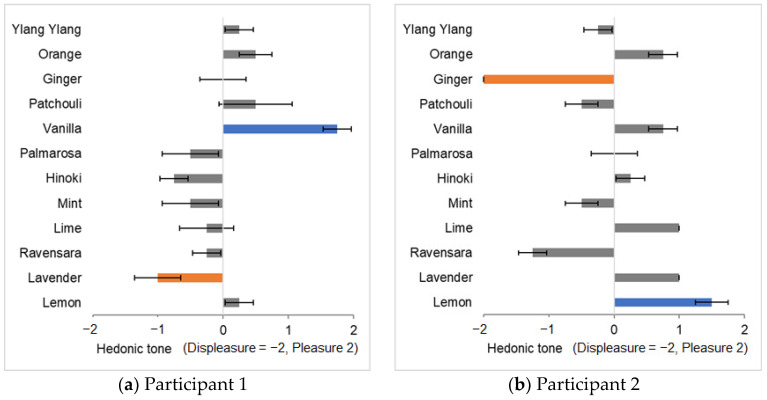
Evaluation results of hedonic tone for fragrance. The pleasant fragrances selected are colored blue, and the unpleasant fragrances selected are colored orange. Also, (**a**) shows the results for participant 1, and (**b**) shows the results for participant 2. The error bars represent the standard error in the two trials.

We selected the appropriate experimental stimuli for each participant by conducting a preliminary survey with 36 colored lights and 12 fragrances. The survey was carried out with eight university students (age 23 ± 1 year). The participants were confirmed to have no olfactory abnormalities or familiarity with essential oils. Yamashita et al. [16] selected olfactory stimuli using a method in which participants selected the most pleasant or unpleasant fragrance among five fragrances. In this case, the pleasure or displeasure associated with each fragrance was relative, making it unclear whether the participants genuinely found the fragrance pleasant or unpleasant. Therefore, in this study, the participants scored each fragrance on a pleasant scale (hedonic tone) to achieve an evaluation that was as absolute as possible. Participants smelled each of the 12 fragrances and rated their hedonic tone on a subjective 5-point scale (−2 to 2). In addition, they described the type of fragrance they perceived. Scent selection data for all participants are shown in Figure S1 in the Supplemental Materials. Each participant completed the survey twice. To minimize the carryover effects from one fragrance to the next, a two-minute break was provided after each fragrance. As in the experiments by Yamashita et al. [16] and Oba et al. [17], participants were not informed of the names of the fragrances, which helped minimize the biases associated with the names and allowed them to evaluate pleasure or displeasure intuitively. Based on these evaluations, two fragrances were selected for each participant (one that they found the most pleasant and another that they found the most unpleasant). Figure 2 shows two examples of the fragrance evaluation results. The horizontal axis represents the pleasantness score, with ‘−2’ indicating displeasure and ‘2’ indicating pleasure. The error bars represent the standard error in the two trials. Next, we conducted a selection survey to determine harmonious and disharmonious color–fragrance combinations. The participants perceived each of the 36 colored lights while smelling each fragrance and rated the harmony between the colored light and the fragrance on an 11-point scale (−5 to 5). Based on these evaluations, we selected the ‘most harmonious colored light (harmonious color)’ and the ‘most disharmonious colored light (disharmonious color)’. Consequently, two fragrances and four colored lights were selected as the experimental stimuli for each participant (Table 1).

There are several points to consider regarding harmony in this study. The first is the criteria that can be used to define what constitutes ‘harmony’. The term ‘harmony’ has often been discussed in the context of intramodal relationships, such as the harmony among multiple sounds or among colors. While recent studies have increasingly focused on harmony across modalities, they have used different definitions of harmony. In this study, harmony was defined as the congruency between the fragrance and colored light. This definition was communicated to the participants before conducting the harmony-rating experiment. However, introspective reports revealed that when the names of the fragrances were not disclosed, participants sometimes failed to accurately imagine the nature of the fragrances, especially that of unpleasant fragrances. As a result, when the names of the fragrances were not provided, it became challenging to evaluate harmony based on the congruency between the fragrance and colored light. In such instances, the participants may have evaluated harmony more metaphorically, focusing on the smoothness and pleasantness of simultaneously perceiving fragrances and colored light. The second point concerns the characteristics of the participants. In this study, we confirmed that the participants were not familiar with essential oils and that some fragrances were difficult for them to identify. However, for individuals who regularly experience fragrances in their daily lives, it may be easier to form concrete imagery and perceive semantic congruency as harmonious, even without knowing the names of the fragrances. The third point pertains to how the mode of presentation of colored light affects the perception of harmony. The psychological states of the participants (such as their emotions) may vary depending on the environment in which the colors are presented, which could influence their subjective evaluation of harmony. This may also apply when harmony is perceived metaphorically. Furthermore, lighting is perceived directly as light, whereas color patches are perceived as reflected light, indicating differences in the mode of perception that may impact the sense of harmony. Therefore, the way in which harmony is perceived may vary depending on the stimulus presentation method.

### 2.4. Experimental Environment

**Figure 3:**
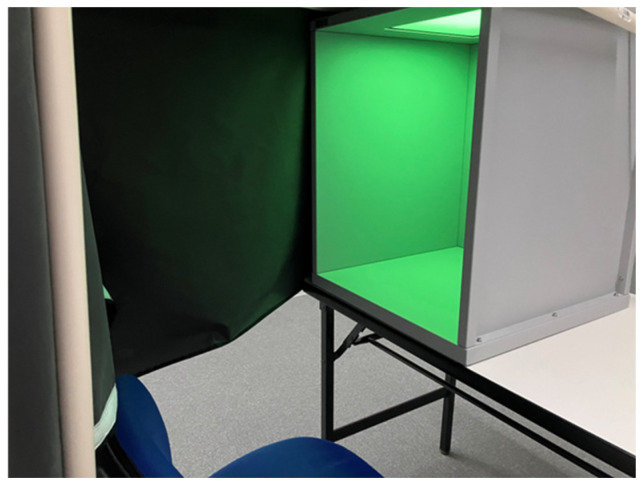
Lighting environment used in the experiment. This figure shows an example of green-colored light.

The participants sat on a chair with an observation box (approximately 45 cm wide and 55 cm high) placed in front of them (Figure 3) and illuminated from above using a spectrally adjustable lighting device (LEDCube, Thousand Lights Lighting (Changzhou) Limited, Changzhou, China). The LEDCube is a lighting environment simulation source capable of reproducing any color by adjusting the power output of monochromatic light. Their performance varied depending on the type of LED incorporated. In this experiment, 14 types of LEDs with different peak wavelengths were used, and the desired colored light was generated by entering the xy values into the software. A spectroradiometer (CL-500A, Konica Minolta, Inc., Tokyo, Japan) was used to verify that the appropriate colored light was emitted. The observation box and the participant were covered with dark curtains to minimize the influence of external stimuli. The illuminance within the observation box was set to 50 lx for all the colored lights, and the experiment was conducted in a dark room.

### 2.5. Experimental Procedure

NIRS was used to measure OFC activation in response to the presentation of colored lights and fragrances selected based on the selection survey. The experiment consisted of the four following conditions: (1) fragrance-only, (2) colored light-only, (3) harmonious crossmodal, and (4) disharmonious crossmodal presentation. The experimental results are summarized in Table 2. Eight university students participated in the selection survey. Each of the four types of experiments was conducted three times for all participants. Furthermore, since Yamashita et al. [16] suggested that the order of the four experimental types could influence ΔoxyHb, the order was randomized to prevent it from affecting the OFC responses. Approximately 30 to 60 s were allocated to each experiment prior to the stimulus presentation in order to stabilize the measurement equipment and brain activity. The stability of brain activity refers to the absence of changes in the oxyHb concentration near the OFC. After confirming stability, the stimuli were presented to the participants for 30 s. The presentation duration was determined based on Yamashita et al. [16], who suggested that a short duration of approximately 10 s did not show a stimulus response, whereas a duration that was too long could lead to reduced participant concentration, thus affecting brain activity. After the presentation, the participants rested for approximately 60 s, during which the response progression was fully monitored. Breaks were taken between presentations, and the well-being of the participants was monitored throughout the experiment.

The experimental procedures for each condition are summarized below.

(1) Fragrance-only
  A sniffing test was conducted using odor bottles. After 30 s of sniffing, the participants were asked to move the bottle away from their nose and return it to the position at their chest. Then, the participants were observed for at least 30 s until their responses stabilized.
(2) Colored light-only
  There was no task for the participants to perform. As soon as the NIRS equipment was stabilized, the experimenter changed the lighting color from white to the desired color, and the participants perceived that color for 30 s. After 30 s, the color was reverted to white. Then, the participants were observed for at least 30 s until their responses stabilized.
(3) Harmonious and (4) disharmonious crossmodal
  A sniffing test using odor bottles was used for the presentation of aroma stimuli. The participant held a bottle containing the fragrance near his/her chest before the stimulus presentation. As soon as the NIRS device was stabilized, the participant brought the bottle close to his/her nose and sniffed the fragrance upon the experimenter’s cue. After 30 s, the experimenter cued the participants to move the bottle away from their nose and change the color back to white. Then, the participants were observed for at least 30 s until their responses stabilized.

### 2.6. Results and Discussion

#### 2.6.1. Difference in Peak Values of oxyHb Concentration in the Left and Right Orbitofrontal Cortex

**Figure 4:**
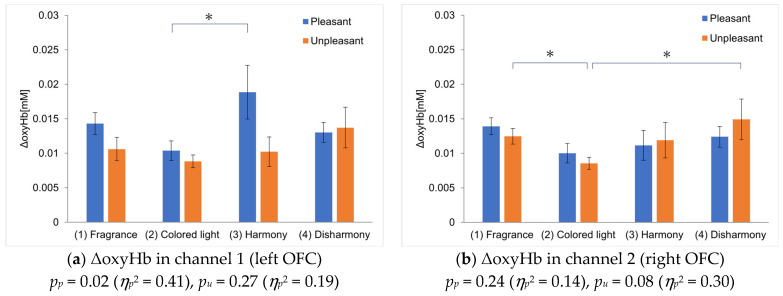
Comparison of ΔoxyHb in the four types of experiments. The asterisks indicate a significance level of 5% in the *t*-test, and the error bars represent the standard error of the mean ΔoxyHb among participants. The same applies to Figure 5, Figure 6 and Figure 7. The error bars indicate the standard error of the mean ΔoxyHb across participants.

We measured OFC activation under four types of stimulus presentations using NIRS. Figure 4 shows the oxyHb levels in the left and right OFC for each experiment. The vertical axis represents the average ΔoxyHb across all participants, and the horizontal axis compares the four experimental conditions. For the colored light-only presentation, the ΔoxyHb values represent the average of the harmonious and disharmonious colors. The error bars indicate the standard error of the mean ΔoxyHb across participants. Additionally, a Smirnov–Grubbs test at a 5% significance level was conducted on all measurement data to remove the outliers, which comprised two data points (approximately 2% of the total data). Figure 4a and Figure 4b show the results for channel 1 and channel 2, representing the left and right OFC, respectively.

For the pleasant fragrance in channel 1, ΔoxyHb was highest during the crossmodal presentation with the harmonious color (Figure 4a). This suggests that the participants experienced greater pleasure when perceiving the fragrance with its harmonious color than when perceiving the fragrance alone. A statistically significant difference was found in OFC activation during the colored light-only presentation and the harmonious crossmodal presentation (* indicates *p* < 0.05). Meanwhile, for the unpleasant fragrance, ΔoxyHb was higher during the crossmodal presentation with the disharmonious color, although the effect was not as pronounced as with the pleasant fragrance. No statistically significant differences were observed among combinations that used unpleasant fragrances. Furthermore, when comparing ΔoxyHb for pleasant and unpleasant fragrances in the four experimental conditions, a substantial difference was observed in the harmonious crossmodal presentation, supporting the inference that the left OFC is associated with pleasant emotions.

For the pleasant fragrance in channel 2, ΔoxyHb increased slightly in the fragrance-only presentation, although no significant differences were observed in the comparisons made with other conditions (Figure 4b). No statistically significant differences were found in channel 2 for the pleasant fragrances for any combination. However, for the unpleasant fragrance, ΔoxyHb was the highest during the crossmodal presentation with the disharmonious color. This suggests that perceiving an unpleasant fragrance with a disharmonious color intensified the unpleasant experience compared with perceiving the fragrance alone. Statistically significant differences were observed between the colored light-only and disharmonious crossmodal presentations and between the fragrance-only and disharmonious crossmodal presentations. Furthermore, the difference in the ΔoxyHb for pleasant and unpleasant fragrances was highest in the disharmonious crossmodal presentation, suggesting that the right OFC is associated with unpleasant emotions. These results differ from those of Yamashita et al. [16], who found that ΔoxyHb was highest when the fragrance was presented alone, suggesting that the order in which the experimental stimuli were presented may influence the findings. Furthermore, the ΔoxyHb results in the colored light-only presentation suggest that perceiving light alone does not activate the OFC as strongly as the fragrance-only or crossmodal presentation, indicating a strong relationship between the OFC and emotions evoked by fragrance.

We have calculated a repeated-measures ANOVA and added values to the captions of Figure 4a,b. Because this study claims that the effect is obtained for a particular combination of two factors, the ANOVA analysis is only a reference, but the numbers generally support the claim in terms of *p*-values and partial *η* squared.

#### 2.6.2. Differences in Difference in Peak Values of oxyHb Concentration Between the Left and Right Orbitofrontal Cortex

Conventional studies have suggested that the left and right OFC may play distinct roles in processing multisensory integration, particularly in evaluating congruent and incongruent sensory inputs [24,25]. Stronger activation of the left OFC under harmonious crossmodal conditions could be related to its role in reward processing and integration of pleasant sensory experiences. The left OFC has been associated with positive affective processing, which could explain its enhanced activity in response to harmonious stimuli.

Conversely, the right OFC’s stronger activation under disharmonious conditions may reflect its involvement in conflict detection, aversive processing, or the evaluation of unexpected stimuli. The right OFC has been linked to the detection of violations of expectations and the processing of conflicting sensory inputs, which may explain its increased activation under disharmonious conditions.

**Figure 5:**
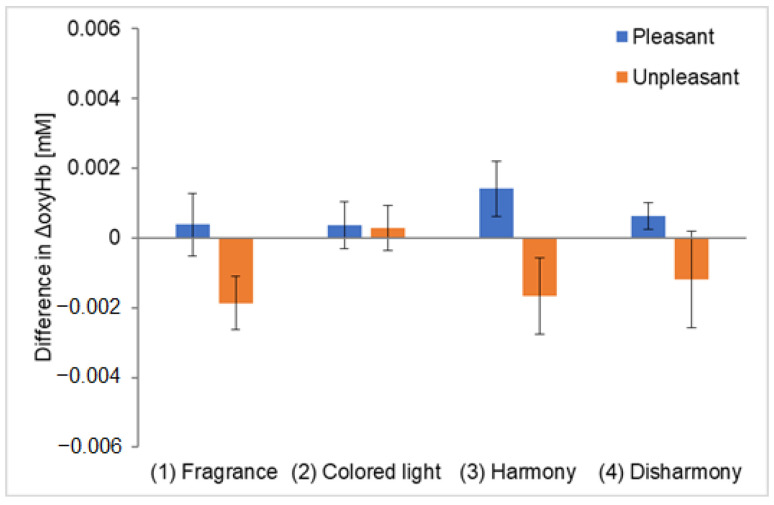
The difference in ΔoxyHb between the left and right OFC. A positive value indicates greater activation in the left OFC, whereas a negative value indicates activation in the right OFC. The error bars represent the standard error of the mean difference in ΔoxyHb across participants.

Figure 5 shows the differences in ΔoxyHb between the left and right OFC. The vertical axis represents the differences in ΔoxyHb between channel 1 and channel 2, calculated using Equation (1):

(1) $$\;Difference\;in\;\Delta oxyHb=\left(\Delta oxyHb\;in\;channel1\right)-\left(\Delta oxyHb\;in\;channel2\right)\;\;$$

A positive value indicates greater activation in channel 1, whereas a negative value indicates greater activation in channel 2. The error bars represent the standard error of the mean difference in ΔoxyHb across participants. As in the ΔoxyHb analysis in Section 2.6.1, outliers were excluded from this analysis as well. For pleasant fragrances, the crossmodal presentation with harmonious colors resulted in the largest channel difference, indicating greater activation of the left OFC. This difference suggests that pleasant emotions were strongest during the harmonious crossmodal presentation, although no statistically significant differences were observed. For unpleasant fragrances, the fragrance-only and crossmodal presentations showed larger response differences, indicating greater activation of the right OFC during these presentations. However, the standard error was higher for the disharmonious crossmodal presentation.

The differences in the patterns observed for pleasant and unpleasant fragrances may be related to participants’ impressions of the fragrances in the selection survey (Section 2.3). As mentioned in Section 2.3, the participants reported being able to imagine an object associated with pleasant fragrances, while impressions of unpleasant fragrances were often vague and unclear. Specifically, although the names of the fragrances were not provided, pleasant fragrances such as orange and lemon often evoked clear mental images, whereas unpleasant fragrances such as ginger and ravensara did not. This suggests that the lack of clarity in participants’ perceptions of unpleasant fragrances may have led to a less accurate matching of harmonious and disharmonious colors with unpleasant fragrances than with pleasant fragrances. This experiment is based on the sensory congruence between fragrance and lighting color, independent of the bias caused by the names of the fragrances, in the analysis of pleasant and unpleasant feelings. However, it is important to note that the degree of imagery evoked by fragrances differs between pleasant and unpleasant odors. Therefore, it is possible that this influenced the OFC’s activation. Additionally, the differences between channels observed during the fragrance-only presentations suggest that displeasure may have intensified because of the participants’ inability to imagine the unpleasant fragrance. Furthermore, the increased standard error during the crossmodal presentations could be due to variations in how the participants perceived harmony with unpleasant fragrances. Most participants reported feeling ‘more pleasant’ when a harmonious color was presented with a pleasant fragrance and ‘more unpleasant’ when a disharmonious color was presented. For unpleasant fragrances, they felt ‘more pleasant’ with a harmonious color and ‘more unpleasant’ with a disharmonious color. However, one participant out of eight reported feeling ‘more unpleasant’ with a harmonious color for the unpleasant fragrance, which may have contributed to the increased error. In this experiment, essential oils were used as olfactory stimuli, which may explain the observed tendency toward judging the fragrances as unpleasant. However, for fragrances that were more unpleasant and easily imaginable by participants, even when presented with a harmonious color, it is possible that they perceived them as unpleasant.

## 3. Experiment II: Analysis of Functional Differences Between the Left and Right Orbitofrontal Cortex

In Experiment I, we conducted the study based on previous findings [20,21,22] suggesting that the left OFC is associated with pleasant emotions, while the right OFC is associated with unpleasant emotions. Our results indicated that in the crossmodal presentation of a pleasant fragrance and its harmonious color, activation of the left OFC enhanced pleasant emotions. Conversely, in the crossmodal presentation of an unpleasant fragrance and its disharmonious color, activation of the right OFC may have enhanced unpleasant emotions. A recent crossmodal study [23] has presented differing views on the functional differences between the left and right OFC in relation to pleasure and displeasure. Specifically, the left OFC is activated when the interaction between colored light and fragrance becomes more harmonious, promoting a crossmodal interaction and becoming dominant in crossmodal perception. In contrast, the right OFC shows a smaller effect from the interaction and is activated when the stimuli are perceived independently, in a more multimodal perception, highlighting this functional difference. Furthermore, studies have shown that even without directly smelling a fragrance, imagining the fragrance or recalling olfactory experiences can activate sensory regions related to olfactory perception, with stronger activation observed in the left OFC [26,27]. Additionally, Broman et al. [28] showed that the right hemisphere is involved in low-level olfactory processing and encoding based on perception, whereas the left hemisphere is associated with higher-order olfactory recognition and semantic interpretation, such as memory and the labeling of fragrances. These findings suggest that the functional differences between the left and right OFC may not be solely determined by ‘pleasure–displeasure’. Instead, another factor may be the extent to which crossmodal perception is enhanced by harmony (higher-order modality-integrated perception), as opposed to stimuli being merely perceived independently due to disharmony (lower-order modality-integrated perception or multimodal perception). In Experiment I, participants were not informed of the names of the fragrances during the stimulus selection phase. However, it has been reported that pleasant fragrances tend to be easier to associate with their corresponding objects, whereas unpleasant fragrances are more difficult to imagine. Given the findings [23,26,27,28], it is possible that this difference in imagery influenced the crossmodal perception of colored light and fragrance. Therefore, in Section 3, we conduct a new NIRS experiment using fragrances that are familiar and easy to imagine for all participants, ensuring that both pleasant and unpleasant fragrances are equally accessible in terms of mental imagery. By analyzing the results, we aim to analyze whether the functional differences between the left and right OFC are influenced not only by the ‘pleasure–displeasure axis’ but also by the ‘crossmodal perception–multimodal perception axis’.

### 3.1. Selection of Experimental Stimuli and Experimental Procedure

We selected the same stimuli as in Experiment I. The same participants (age 23 ± 1 year) participated in this experiment. We selected familiar fragrances from the 12 essential oils used in Experiment I. Eight fragrances were selected: orange, ginger, vanilla, hinoki, mint, lime, lavender, and lemon. The ‘most pleasant’ and ‘most unpleasant’ fragrances were selected from this list following the same procedure as in Section 2.3. Scent selection data for all participants are shown in Figure S2 in the Supplemental Materials. This survey revealed that knowledge of the name of the fragrance affected the subjective hedonic tone judgment of many participants. This finding indicates that the imagery evoked by the names of fragrances significantly influences the pleasant–unpleasant evaluation of the fragrance. Harmonious and disharmonious colors were identified for the selected fragrances. As in Experiment I, the participants were informed of the definition of harmony prior to performing the evaluation experiment using the same experimental conditions as described in Section 2.5. However, because they were aware of the names of the fragrances, we inferred that the participants evaluated harmony based on the semantic congruency between the fragrance and colored light.

### 3.2. Results and Discussion

#### 3.2.1. Difference in Peak Values of oxyHb Concentration in the Left and Right Orbitofrontal Cortex

**Figure 6:**
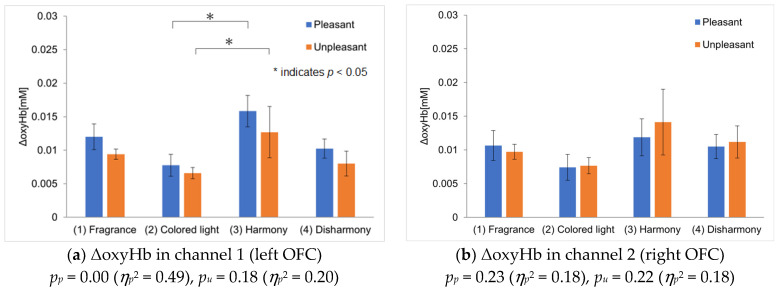
Comparison of ΔoxyHb between four experimental conditions (in Experiment II). The error bars indicate the standard error of the mean ΔoxyHb of the participants.

NIRS was used to measure OFC activation in response to the four types of presentations. The oxyHb values for each experiment are shown in Figure 6; the vertical axis represents the average ΔoxyHb across all participants, while the error bars indicate the standard error of the mean ΔoxyHb of the participants. The ΔoxyHb for the colored light-only presentation is the average value obtained from responses to harmonious and disharmonious colors. Five outliers were removed using the Smirnov–Grubbs test at a 5% significance level, accounting for approximately 5% of the total data. Figure 6a and Figure 6b show the analysis results for channel 1 and channel 2, corresponding to the left and right OFC, respectively. The ΔoxyHb measured on channel 1 was the highest during the crossmodal presentations of pleasant fragrances with harmonious colors, similar to the results of Experiment I (Figure 6a). However, ΔoxyHb was also high during the crossmodal presentations of unpleasant fragrances with harmonious colors, a result different from that seen in Experiment I. This suggests that the participants might have imagined harmony more effectively for unpleasant fragrances in Experiment II, leading to dominant crossmodal perception and activation in the left OFC. Statistically significant differences were observed between the colored light-only and harmonious crossmodal presentations for both pleasant and unpleasant fragrances. For channel 2, while ΔoxyHb was slightly higher during the harmonious crossmodal presentation with pleasant fragrances, the difference was not significant (Figure 6b). For unpleasant fragrances, ΔoxyHb peaked during a harmonious crossmodal presentation; however, in contrast to Figure 6a, the error was large, and no statistically significant differences were observed between conditions. We have calculated a repeated-measures ANOVA and added values to the captions of Figure 6a,b.

#### 3.2.2. Differences in Difference in Peak Values of oxyHb Concentration Between the Left and Right Orbitofrontal Cortex

**Figure 7:**
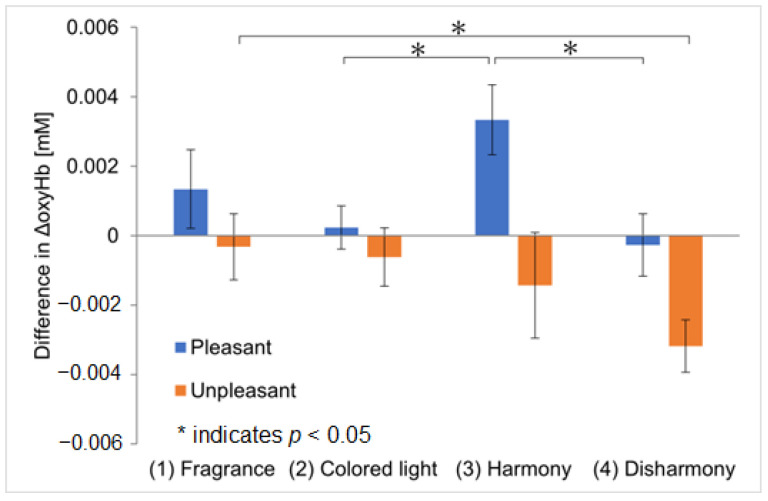
The difference in ΔoxyHb between the left and right OFC (in Experiment II). The error bars represent the standard error of the mean ΔoxyHb difference among the participants.

Figure 7 shows the results of the analysis of the ΔoxyHb differences between the left and right OFC, the vertical axis representing the difference in ΔoxyHb between channel 1 and channel 2, calculated using Equation (1). The error bars represent the standard error of the mean ΔoxyHb difference among the participants. The outliers described in Section 3.2.1 were excluded from this analysis as well. For pleasant fragrances, the largest channel difference was observed during the crossmodal presentations with harmonious colors, confirming greater activation in the left OFC. This suggests that the greatest sense of pleasure was evoked during the crossmodal presentation with harmonious colors, yielding results similar to those of Experiment I. However, in contrast to Experiment I, significant differences were found between the ‘colored light-only and harmonious crossmodal’ and ‘harmonious and disharmonious crossmodal’ presentations, suggesting that knowing the names of the fragrances may enhance pleasure or create dominant crossmodal effects. For unpleasant fragrances, the difference in responses increased during the disharmonious crossmodal presentations, with greater activation in the right OFC. This result differs from that of Experiment I, indicating that the participants might have perceived unpleasant fragrances and disharmonious colored light as individual stimuli, leading to the activation of the right OFC. Therefore, Experiment II suggests that the left and right OFC were influenced not only by a ‘pleasure–displeasure axis’ but also by a ‘crossmodal perception–multimodal perception axis’.

## 4. Additional Experiment: Correlation Between Hedonic Tone and Degree of Harmony in Subjective Evaluation

The results of Experiment I suggest that when a pleasant fragrance and its harmonious color are crossmodally perceived, the left OFC is activated, potentially enhancing pleasant emotions. In contrast, when an unpleasant fragrance and its disharmonious color are crossmodally perceived, the right OFC is activated, potentially enhancing unpleasant emotions. In our experiments in the previous sections, NIRS was used to objectively analyze the recall of pleasant and unpleasant emotions while considering the functional differences of the left and right OFC, as reported in the literature [20,21,22]. However, it was not possible to verify whether the participants judged pleasure or displeasure subjectively. Therefore, as an additional experiment, the relationship between the subjective hedonic tone and the degree of harmony in the crossmodal perception of colored light and fragrance was investigated.

**Figure 8:**
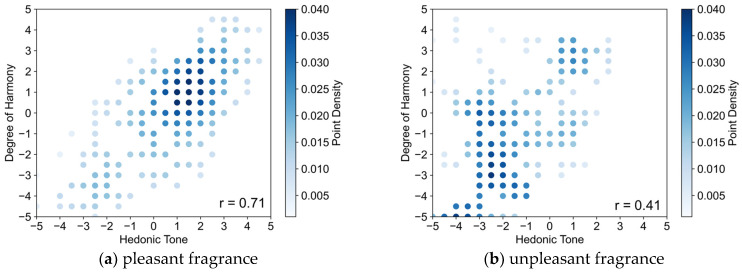
Correlation between hedonic tone and degree of harmony.

First, an experiment was conducted where the subjective hedonic tone of the crossmodal perception of two types of pleasant and unpleasant fragrances selected in 3.2 and 36 lighting colors generated in 3.1 was evaluated. There were six of the eight participants who participated in Experiment I (age 23 ± 1 year). The participants simultaneously perceived the colored light and fragrance and rated the hedonic tone of the experience on an 11-point scale. The experimental environment was the same as in Section 2.4. Using the evaluation results of hedonic tone and the degree of harmony evaluation results from Section 2.3, a scatter plot showing their correlation is shown in Figure 8a shows the results for the pleasant fragrance, and (b) shows the results for the unpleasant fragrance. The vertical axis represents the degree of harmony, and the horizontal axis represents the hedonic tone. Additionally, the closer the score is to ‘5’, the more harmonious or pleasant it is, while the closer it is to ‘−5’, the more disharmonious or unpleasant it is. The correlation coefficients for each are noted in the lower right of the scatter plots. The density of points due to overlapping is shown using a heatmap based on Kernel Density Estimation.

In the case of the pleasant fragrance shown in Figure 8a, a high positive correlation of 0.71 was confirmed between the hedonic tone and harmony in the crossmodal perception of colored light and fragrance. This indicates that for the pleasant fragrance, colored lights with higher harmony were perceived as more pleasant, while those with lower harmony suppressed or induced displeasure. This result supports the findings of Experiment I, where the left OFC was activated when a pleasant fragrance and its harmonious color were crossmodally perceived, enhancing pleasant emotions. In contrast, for the unpleasant fragrance shown in Figure 8b, the correlation coefficient was 0.41, which is lower than for the pleasant fragrance but still indicates a positive correlation. This suggests that for the unpleasant fragrance, colored lights with lower harmony were perceived as more unpleasant, while those with higher harmony suppressed or induced pleasure. This result also supports the findings of Experiment I, where the right OFC was activated when an unpleasant fragrance and its disharmonious color were crossmodally perceived, enhancing unpleasant emotions. The slightly lower correlation for the unpleasant fragrance can be attributed to the difference in how one participant perceived harmony, as mentioned in Section 2.6.2. This participant perceived the combination of the unpleasant fragrance and its harmonious color as more unpleasant, which may have influenced the negative correlation. When excluding this participant, the correlation coefficient was 0.55.

In Experiment II, it was suggested that the functional differences between the left and right OFC are also influenced by the ‘crossmodal perception–multimodal perception axis’. Focusing on the degree of harmony between the colored light and fragrance, it was found that when the degree of harmony is high, semantic crossmodal perception becomes dominant, resulting in higher-order modality-integrated perception. In contrast, when the degree of harmony is low, the stimuli are not semantically linked and are processed merely as independent stimuli, resulting in lower-order modality-integrated perception. Based on this, it can be inferred that for the pleasant fragrance, higher harmony in the colored light enhances pleasant emotions, indicating that crossmodal perception leads to pleasure. Conversely, for the unpleasant fragrance, lower harmony in the colored light enhances unpleasant emotions, suggesting that multimodal perception leads to displeasure. Therefore, it can be concluded that the ‘pleasant–unpleasant axis’ and the ‘crossmodal perception–multimodal perception axis’ are interrelated in the functional differences between the left and right OFC.

The difference between crossmodal and multimodal perception can be viewed neuroscientifically as a difference in the following actions [29].

Crossmodal perception involves interactions between different sensory modalities, wherein information from one sense influences the perception of another. This process may engage multiple brain regions, such as:

- The orbitofrontal cortex and insula, which integrate sensory inputs to form coherent perceptual experiences.
- The superior temporal sulcus, which plays a key role in integrating audiovisual stimuli.
- The intraparietal sulcus, which coordinates attentional resources across the senses.

The piriform cortex and visual association area information are linked, and the sense of smell is enhanced by vision. Crossmodal processing might be characterized by dynamic interactions between these regions and often requires more extensive neural integration than unimodal processing.

By contrast, multimodal perception refers to the parallel processing of multiple sensory inputs without necessarily integrating them into a unified experience. This process often relies more on independent sensory-specific brain regions. Primary sensory cortices (e.g., the visual, entorhinal, and piriform cortices) process different sensory inputs separately. Higher-order association areas may still interact, but the level of integration is lower than that of crossmodal perception.

## 5. Conclusions

This study focused on the lateralization of brain function in the left and right OFC by analyzing the pleasure–displeasure evoked by harmony between colored light and fragrance. Two experiments were conducted, each following the processes of stimulus generation, selection, and NIRS measurement during stimulus presentation. In Experiment I, 36 colored lights and 12 fragrances were used as stimuli. A selection survey was used to have participants rate the hedonic tones of 12 fragrances without knowing their names—of which the most pleasant and unpleasant fragrances were selected. The participants then rated the degree of harmony between the selected fragrances and colored lights, leading to the selection of ‘harmonious’ and ‘disharmonious’ color–fragrance combinations. Each participant was presented with two different fragrances and four colored lights as the experimental stimuli. Based on these selected stimuli, NIRS measurements of OFC activity were performed under the four following presentation conditions: fragrance-only, colored light-only, harmonious crossmodal, and disharmonious crossmodal presentations. The NIRS device measured changes in the oxyHb concentrations in the left and right OFC. Moving averages were applied to the measured data, and the difference in oxyHb concentration (ΔoxyHb) before and after stimulus presentation was calculated as a response to each stimulus. We found that the ΔoxyHb associated with channel 1 was the largest during the crossmodal presentation with harmonious colors. This suggests that the participants felt more pleasant when they perceived the harmonious color simultaneously with the pleasant fragrance rather than when only smelling the pleasant fragrance. The ΔoxyHb measured on channel 2 was the largest during the crossmodal presentation with disharmonious colors. This suggests that participants felt more displeasure when they perceived the disharmonious color simultaneously with the unpleasant fragrance rather than when they only smelled the unpleasant fragrance. In Experiment II, eight familiar fragrances were selected from the original list of twelve fragrances, and the same procedures as in Experiment I were followed to select the ‘most pleasant’ and ‘most unpleasant’ fragrances, as well as the ‘harmonious’ and ‘disharmonious’ colors associated with them. The NIRS measurements in Experiment II showed that ΔoxyHb was highest in channel 1 for the pleasant and unpleasant fragrances in the harmonious crossmodal presentation, suggesting that the left OFC is more active when crossmodal effects are dominant. An additional experiment showed a positive correlation between hedonic tones and the degree of harmony, indicating that harmonious colors evoke pleasure, whereas disharmonious colors evoke displeasure. This suggests that the lateralization of brain function in the left and right OFC arises from the interaction between the ‘pleasure–displeasure axis’ and ‘crossmodal perception–multimodal perception axis’.

Although our work is rooted in experimental psychophysics, its significance extends to the field of sensing technology, particularly to the application and optimization of NIRS for studying multisensory interactions. To better highlight this aspect, we have refined our discussion to emphasize the following points:

1. Innovation in Sensing Methodology

Our study employed NIRS in a novel way to examine the psychophysical correlates of crossmodal interactions between light and olfactory stimuli. Unlike conventional applications of NIRS, we specifically investigated its use for detecting subtle hemodynamic responses associated with sensory integration.

2. Contribution to Sensor-Based Research

Although we did not design a new sensor, our approach introduces an innovative application of existing sensing technology to a relatively unexplored research domain. By demonstrating how NIRS can be effectively utilized in multisensory studies, our study lays the groundwork for further developments in sensor-based neuroscience and biomedical engineering.

Future studies should explore different methods for analyzing OFC responses. In this study, moving averages were applied to the measurement data, and oxyHb was used as the response to each stimulus. Further analyses that focus on deoxygenated hemoglobin using GLM-based analyses or a combination of these methods may allow for a more objective analysis of the physiological responses. Furthermore, the fragrances used in this study were essential oils, which may have limited the range of displeasure tested in this analysis. Using more intense and unpleasant fragrances could potentially reveal a more distinct lateralization of brain function in the OFC. Moreover, as mentioned in Section 2.3, differences in the color presentation method may influence the harmony perceived by participants; this requires experimental demonstration. While the sample size of eight participants is relatively small, it is required for larger follow-up studies to validate our results. Although gender differences were not a primary focus of this study, we recognize their potential influence on sensory perception and neural responses. Also, future research should incorporate a more diverse sample to examine potential gender-related variations. A recent study has proposed a method for understanding odor descriptions in multimodal contexts, where odor information is communicated indirectly through text and images [30]. An analysis of the relevance of those multimodal studies is also an issue.

## Figures and Tables

**Table 1:** Type of selected stimulus.

Fragrances (2 Types)	Colored Lights (4 Types)
pleasant fragrance	harmonious color
	disharmonious color
unpleasant fragrance	harmonious color
	disharmonious color

**Table 2:** Breakdown of the four types of experiments.

Experiment (1): Fragrance-Only	Experiment (2): Colored Light-Only	Experiment (3): Harmonious Crossmodal	Experiment (4): Disharmonious Crossmodal
pleasant fragrance	harmonious color	pleasant–armonious	pleasant–disharmonious
	disharmonious color		
unpleasant fragrance	harmonious color	unpleasant–harmonious	unpleasant–disharmonious
	disharmonious color		

## Data Availability

Data are contained within the article.

## Supplementary Materials

The following supporting information can be downloaded at: https://www.mdpi.com/article/10.3390/s25072230/s1, A Results of Fragrance Selection Survey for all participants.

## Abbreviations

The following abbreviations are used in this manuscript:

OFC	Orbitofrontal Cortex
NIRS	Near-Infrared Spectroscopy
fMRI	functional Magnetic Resonance Imaging
PCCS	Practical Color Coordinate System
oxyHb	Oxygenated Hemoglobin
