# Supplementary material for: Analysis of Pleasure and Displeasure in Harmony Between Colored Light and Fragrance by the Left and Right OFC Response Differences
Source: Sensors (Basel). 2025 Apr 2;25(7):2230. doi: 10.3390/s25072230 (PMC11991507; doi:10.3390/s25072230)
Supplement: Supplementary file 1 [file sensors-25-02230-s001.zip › sensors-3504372-supplementary.pdf]

A Results of Fragrance Selection Survey for all participants

S.1 Section 3

Figure S1 shows the scent selection data for all participants. The most pleasant scents are colored blue, and the most unpleasant scents are colored orange.

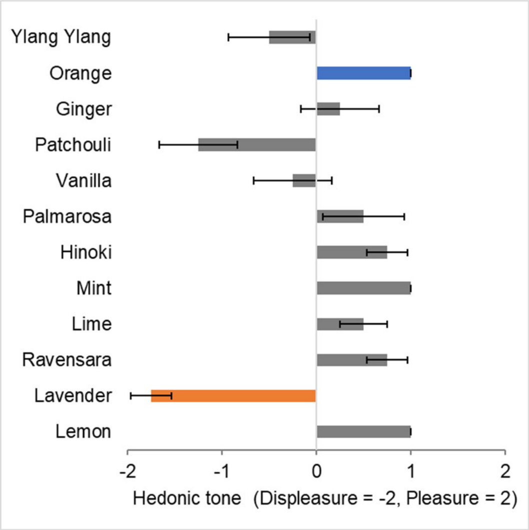

(a) No.1

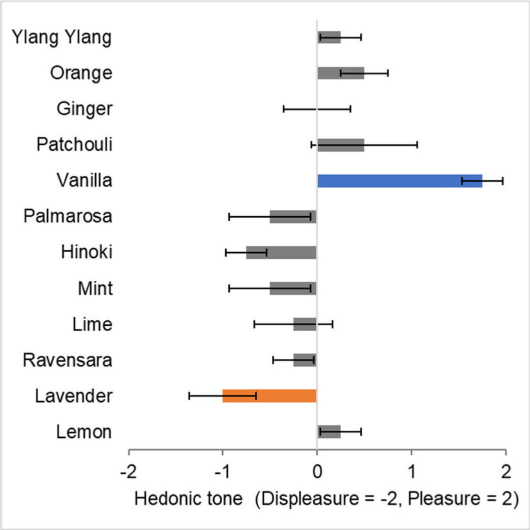

(b) No.2

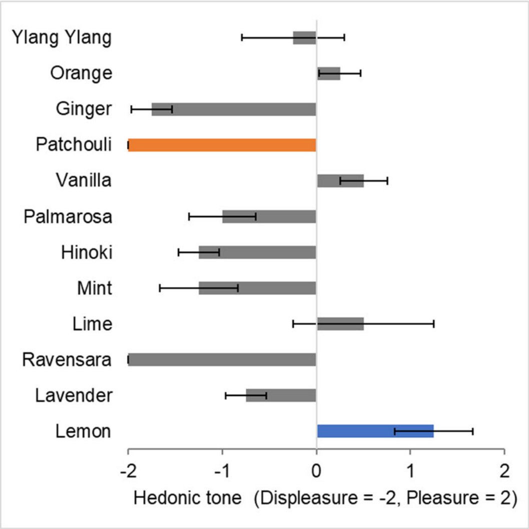

(c) No.3

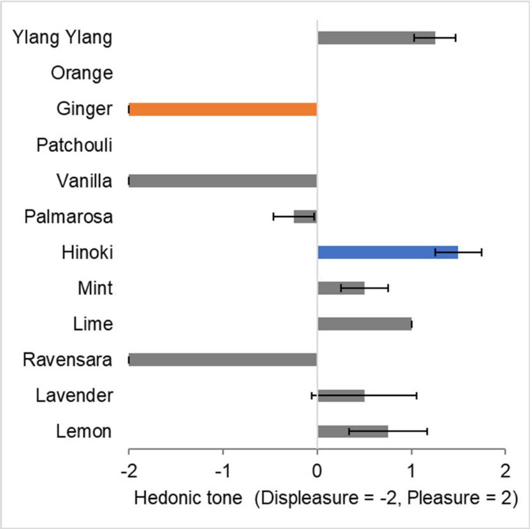

(d) No.4

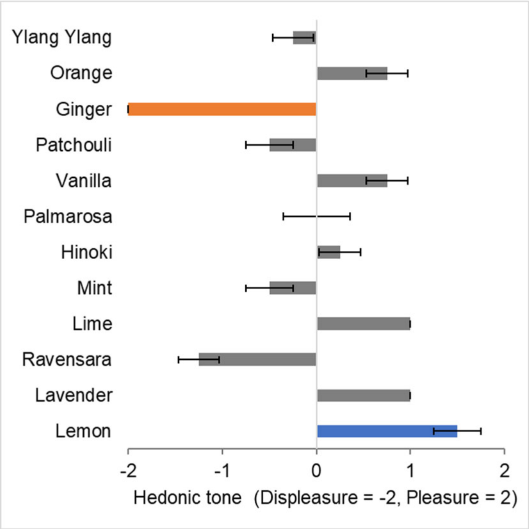

(e) No.5

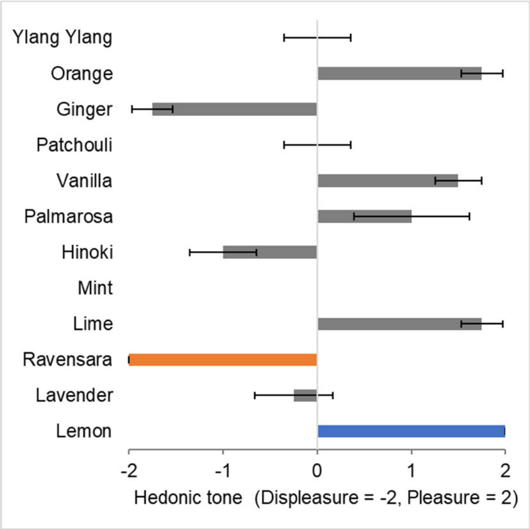

(f) No.6

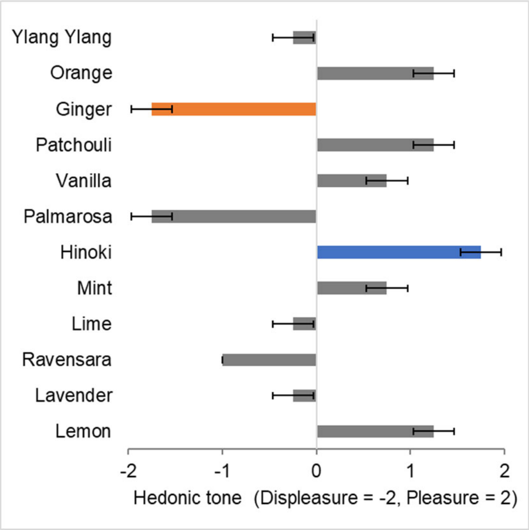

(g) No.7

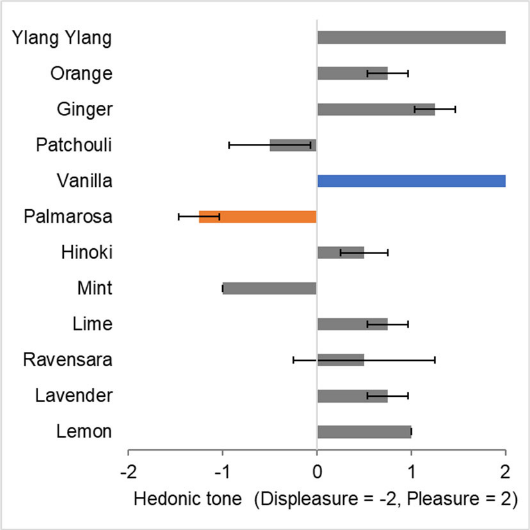

(h) No.8

Figure S1

S.2 Section 4

Figure S2 shows the scent selection data for all participants. The most pleasant scents are colored blue, and the most unpleasant scents are colored orange.

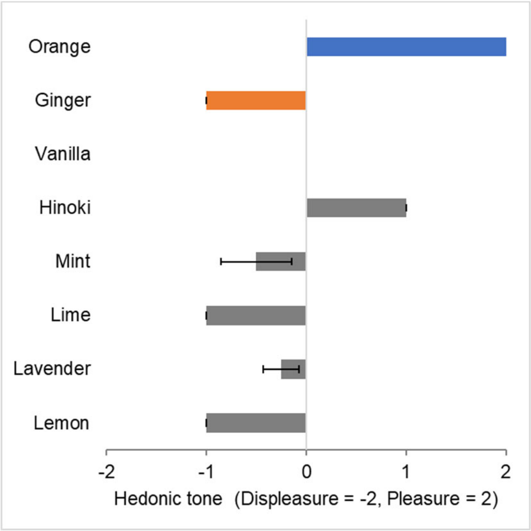

(a) No.1

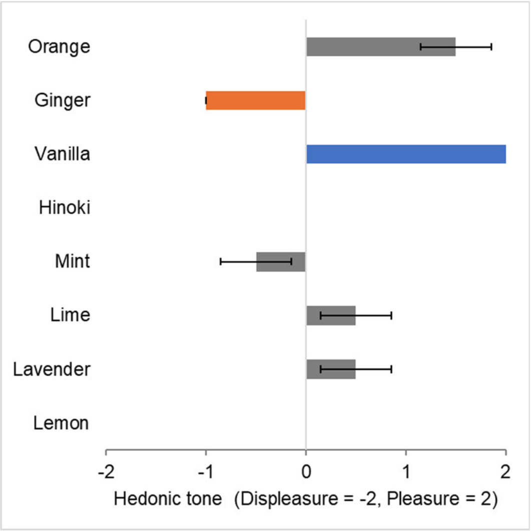

(b) No.2

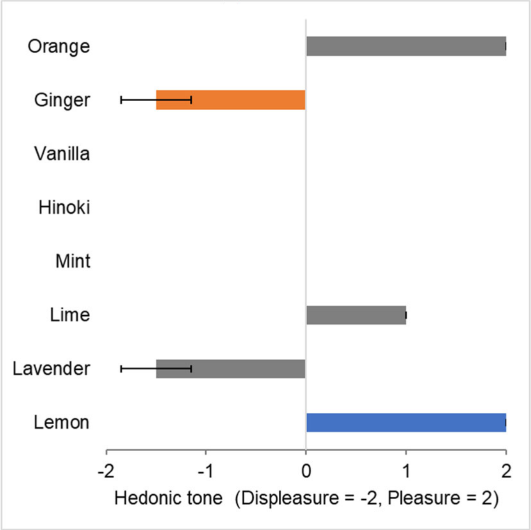

(c) No.3

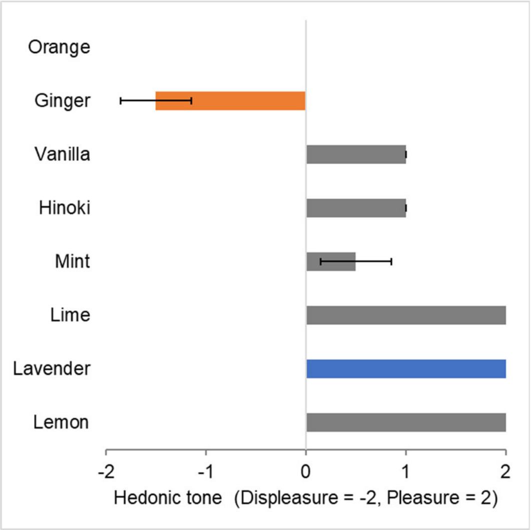

(d) No.5

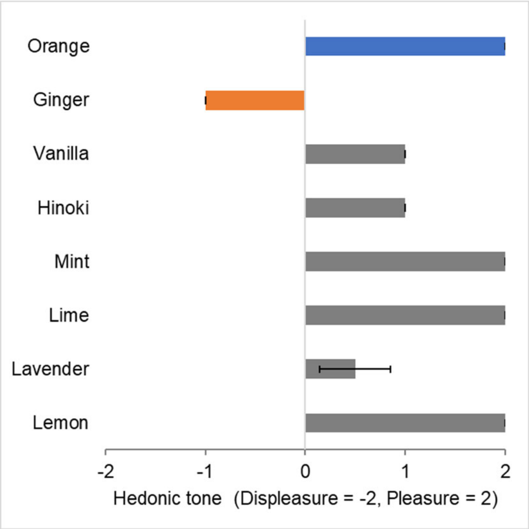

(e) No.6

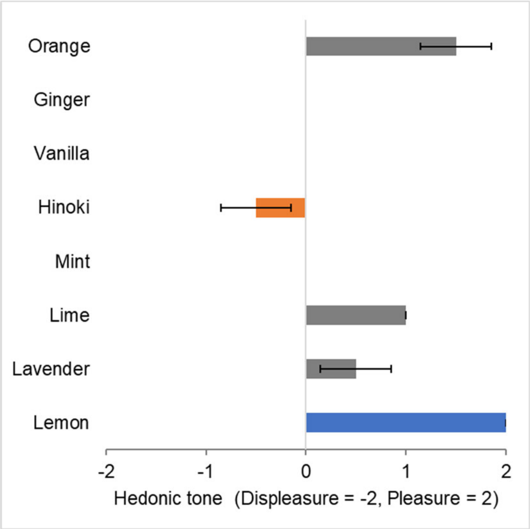

(f) No.9

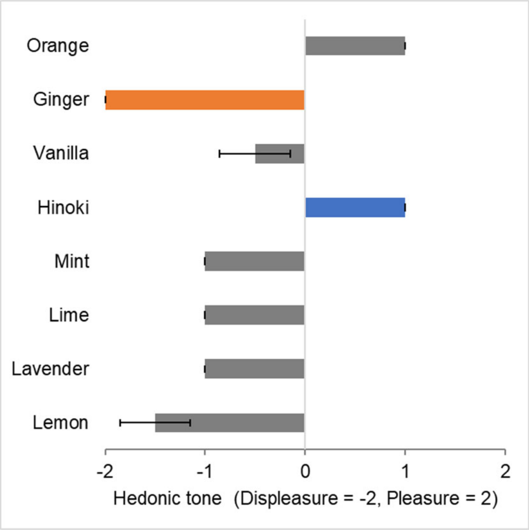

(g) No.10

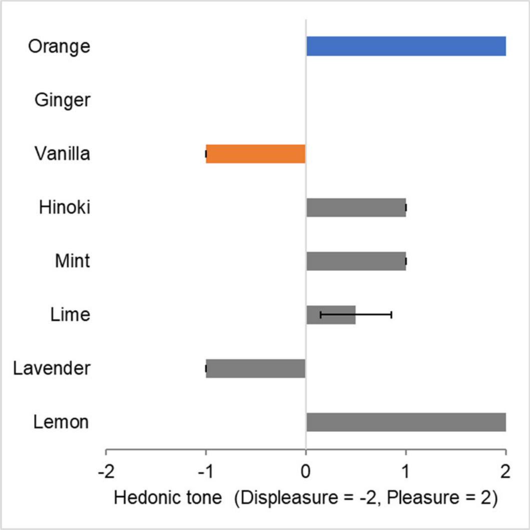

(h) No.11

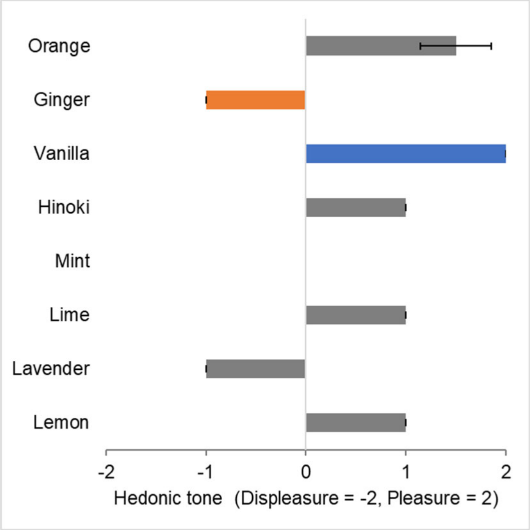

(i) No.12

Figure S2
